# Correlation Analyses Reveal a Limited Role of Transcription in Genome-Wide Differential MicroRNA Expression in Mammals

**DOI:** 10.3389/fgene.2018.00154

**Published:** 2018-05-01

**Authors:** Xiaoxiao Zhang, Siling Hu, Jia Su, Zixuan Xie, Wenjing Li, Yan Zeng

**Affiliations:** Department of Zoology, College of Life Sciences, Nanjing Agricultural University, Nanjing, China

**Keywords:** miRNA, transcription, pri-miRNA, miRNA processing, correlation analysis, ENCODE datasets

## Abstract

Transcription initiates the cascade of gene expression and is often assumed to play a predominant role in determining how much gene products are ultimately expressed. The relationship between mRNA levels and protein levels has been studied extensively to reveal the degrees of transcriptional and post-transcriptional regulation of protein expression. The extent to which transcription globally controls the differential expression of non-coding RNAs, however, is poorly defined. MicroRNAs (miRNAs) are a class of small, non-coding RNAs whose biogenesis involves transcription followed by extensive processing. Here, using hundreds of datasets produced from the ENCODE (Encyclopedia of DNA Elements) project we calculated the correlations between transcriptional activity and mature miRNA expression in diverse human cells, human tissues, and mouse tissues. While correlations vary among samples, most correlation coefficients are small. Interestingly, excluding miRNAs that were discovered later or weighting miRNA expression improves the correlations. Our results suggest that transcription contributes only modestly to differential miRNA expression at the genome-wide scale in mammals.

## Introduction

How gene expression is regulated at the global scale is among the most intensely studied subjects in genomics (Vogel and Marcotte, 2012; Liu et al., 2016). Transcription, splicing, cleavage, modification, and degradation all modulate RNA expression, and protein expression is likewise determined by mRNA translation, protein modification, and degradation. The correlations between mRNA levels and protein levels in various model organisms and systems have been investigated (Gygi et al., 1999; Ghaemmaghami et al., 2003; Beyer et al., 2004; Brockmann et al., 2007; Schmidt et al., 2007; Wu et al., 2008; de Sousa Abreu et al., 2009; Maier et al., 2009; Lundberg et al., 2010; Vogel et al., 2010; Ghazalpour et al., 2011; Schwanhausser et al., 2011; Ponnala et al., 2014; Shaik et al., 2014; Jovanovic et al., 2015; Edfors et al., 2016), with recent estimates that mRNA levels can explain over 80% of the variance in protein levels (Li et al., 2014; Csardi et al., 2015). Because the contribution by mRNA degradation has always been shown to be minor, transcription (including processing) is considered a dominant step in controlling protein expression (Li et al., 2014).

Besides proteins, cells also produce a large number of non-coding RNAs, e.g., ribosomal RNAs, transfer RNAs, small nuclear RNAs, small nucleolar RNAs, MicroRNAs (miRNAs), small interfering RNAs, piwi-interacting RNAs, and long non-coding RNAs (lncRNAs). In contrast to protein expression, how transcription regulates non-coding RNA levels at the genome-wide scale has not been examined in detail. This is paradoxical, as some of the RNA species have been well characterized, and it is easier to quantify RNAs than proteins. Nonetheless, analyzing non-coding RNAs at a large scale does face a few technological challenges. One is that certain RNA classes are encoded by multiple genes, sometimes with complex genomic structures. Another is that prevailing RNA-seq techniques typically yield short sequence reads that often do not adequately distinguish between RNAs such as small nucleolar RNAs and lncRNAs and their initial transcripts or processed intermediates. Moreover, lncRNAs are mostly ill-defined but closely mimic mRNAs or their precursors. The biogenesis of ribosomal RNAs, transfer RNAs, and small nuclear RNAs is coupled to the physiological status of a cell and constrained by the requirement for stoichiometric complex formation (Jinks-Robertson et al., 1983; Mangin et al., 1985; Paule and White, 2000). Still, the global regulatory mechanisms of other RNAs such as miRNAs remain to be elucidated.

miRNAs consist of a large family of approximately 22-nucleotide-long RNAs that inhibit target gene expression in metazoans (Tran and Hutvagner, 2013; Hammond, 2015). miRNA genes are typically transcribed by RNA polymerase II (Pol2) to generate the long, primary miRNA transcripts or pri-miRNAs, which are indistinguishable from and/or overlap with (known) mRNAs, heterogeneous nuclear RNAs (pre-mRNAs), or lncRNAs. The RNAs subsequently undergo a series of processing steps, including cleavage by DROSHA and DICER, to produce mature miRNAs, although some miRNAs can forego the requirement for DROSHA or DICER during their biogenesis (Tran and Hutvagner, 2013).

Like mRNAs and proteins, miRNAs vary widely in expression levels in cells. Transcription is commonly presumed by default to be the major driving force in differential RNA expression, as in the case of protein production, but direct evidence that it regulates miRNA expression at the global scale is lacking. A study in 3T9 mouse fibroblasts reported that transcription highly correlated with miRNA expression (Marzi et al., 2016). On the other hand, while miRNA expression has been shown to be regulated by DROSHA processing (Feng et al., 2011; Conrad et al., 2014), investigation of a handful of human cell lines found transcription activity correlated only weakly, if at all, with mature miRNA levels (Graves and Zeng, 2012; Conrad et al., 2014). The above studies employed only a small sample size, so the results might be affected by unequal genomics data quality or idiosyncrasy of the cell lines that were analyzed. As transcription has been traditionally considered a major determinant of gene expression, in this study, we decided to investigate its contribution comprehensively, by examining how transcriptional activity correlated with miRNA expression in a broad range of human and mouse cell and tissue samples, taking advantage of a large collection of RNA-seq and ChIP-seq datasets from the ENCODE (Encyclopedia of DNA Elements) consortium (ENCODE Project Consortium, 2012). ENCODE datasets were chosen because the ENCODE project has used well documented, characterized, and standardized materials, techniques, and procedures to generate the most complete, easy to access, thousands of processed datasets, including many replicates, with reportedly good data quality. miRNAs were chosen as the subject because miRNAs are typically of a single gene copy, and mature miRNAs can be differentiated from longer transcripts by standard RNA-seq, thereby offering a facile system to study how transcription regulates the expression of non-coding RNAs at the genome level. By inference one might also be able to gain insights into the relative contribution to miRNA abundance by DNA transcription and RNA processing. As for our hypothesis, we expected that transcription contributes to differential miRNA expression, producing positive correlation coefficients, and the higher the coefficients, the greater the contribution.

## Materials and Methods

All datasets were downloaded from the ENCODE portal^1^. To maintain consistencies in data processing and analyses, for Pol2 (POLR2AphosphoS2 for A549 and HeLa-S3 cells, POLR2A for all other samples) ChIP-seq results we used only those samples with available processed data in the bed format; for RNA-seq, including miRNA-seq, small RNA-seq, total RNA-seq, and polyA RNA-seq, we downloaded only processed data with the gene quantifications tsv output. In other words, we extracted only the simplest, most annotated and processed data. We used GM12878, one of the best tested cell lines by ENCODE, to represent the GM series of cells. We downloaded all ENCODE datasets that met these criteria as on March 31, 2017.

In ENCODE datasets, miRNA expression includes both the 5p and 3p miRNA species and, hence, represents complete miRNA production from any particular gene locus. Because miRNA genes are poorly characterized, we acquired human and mouse precursor miRNAs (pre-miRNA) genome information from the miRBase (Kozomara and Griffiths-Jones, 2014), arbitrarily extended a set distance at both the 5′ and 3′ directions, e.g., 1, 2, 5, 10, or 20 kb, and then used the resulting segments to search for overlapping Pol2 ChIP-seq and RNA-seq signals in the ENCODE datasets. The miRNA genome information from miRBase does not uniformly correspond to pre-miRNAs, but such minor variations unlikely affect our analyses and outcomes. In a separate analysis, when human and mouse pri-miRNAs had been experimentally determined (Chang et al., 2015), we would directly search their overlaps with RNA-seq data, or extend a certain distance as mentioned above from both the 5′ and 3′ ends of the pri-miRNAs, and then search for overlaps with Pol2 ChIP-seq data. Notably, the median length of human pri-miRNAs is approximately 41 kb, mouse 36 kb (Chang et al., 2015). The Galaxy website^2^ was used to find overlaps and join different datasets into single files for correlation studies (Afgan et al., 2016). For ChIP-seq, we considered Pol2 peaks on both DNA strands. For RNA-seq, we considered transcripts from only the miRNA-coding strands. Because mRNAs (including non-coding RNAs, unless specified otherwise) are long molecules yet will score “positive,” in theory, with only a one-nucleotide overlap, it is possible that for certain RNA species most sequencing signals might lie outside of the extended miRNA segments, but due to our incomplete knowledge of miRNA gene structures, the consideration of such mRNAs is reasonable. The resulting Galaxy files were downloaded, and Excel (Microsoft Corp.) used to further process the data.

For weighting factors, three parameters from miRBase (Kozomara and Griffiths-Jones, 2014) were used. The first is total deep sequencing reads of the whole miRNA stem-loop, the second is its normalized reads per million, and the third is the sequencing reads of the mature miRNA (both the 5p and 3p).

SPSS 17 (IBM Corp.) was used to compare gene expression and calculate the Spearman rank correlation coefficients and Pearson correlation coefficients and their two-sided *p*-values when possible. A *p* < 0.05 was considered statistically significant. For ChIP-seq studies, all the ChIP-seq signals corresponding to the same miRNA segment were added and compared to the miRNA expression. Because a miRNA might be linked to multiple genes/mRNAs, to compute correlations to mRNA expression, we had used the sums of all the mRNA Fragments Per Kilobase Million signals, or the maximal signal. Both treatments gave very similar results, so results with the summation method are presented here. To estimate experimental noise, overlapped data were used to compute Spearman correlation coefficients between miRNA expression and ChIP-seq signals, between miRNA expression and mRNA expression, and between the duplicate datasets (Csardi et al., 2015).

Hierarchical clustering was performed using the Cluster 3.0 program (Eisen et al., 1998; de Hoon et al., 2004), and results visualized by TreeView (Saldanha, 2004).

## Results

### Correlations Between Transcriptional Activity and miRNA Expression in Human Cells

Most miRNAs are transcribed by Pol2, so Pol2 binding as determined by ChIP-seq experiments approximates transcriptional activity in miRNA genes. But because Pol2 datasets are relatively limited, we also used the expression of mRNAs as a proxy for transcription around miRNA genes. For simplicity, unless specified otherwise, mRNAs referred hereafter also include non-coding RNAs, e.g., lncRNAs, many of which have already been annotated as pri-miRNAs in ENCODE datasets. We downloaded all the human and mouse ENCODE datasets that met our requirements (see section “Materials and Methods”) for correlation analyses. In total, we compared ChIP-seq and miRNA expression data in 11 human cells and 10 human tissues or organs, mRNA and miRNA expression in 41 human cell samples (including immortal cell lines, primary cells, stem cells, and differentiated cells), 62 human tissues, and 40 mouse tissue samples. When there were replicates, we randomly selected one of them for correlation studies, and all such datasets are listed in Supplementary Tables 1–3.

**FIGURE 1:**
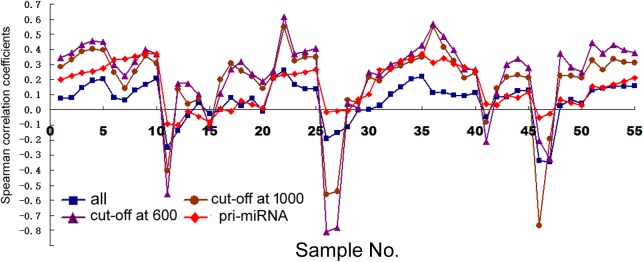
Spearman correlations between miRNA expression and Pol2 ChIP-seq signals in human cells. Corresponding to the data of Supplementary Table 4, the *y*-axis shows the correlation coefficients, and the *x*-axis represents the 11 cell samples, each with 1, 2, 5, 10, and 20 kb genomic extensions from both 5′ and 3′ ends of the pre-miRNAs or pri-miRNAs used for overlap searches. Different symbols and colors represent various miRNA sample sets: all the miRNAs in miRBase (“all”), miRNAs with numbers below 1000 (“cut-off at 1000”), miRNAs with numbers below 600 (“cut-off at 600”), and miRNAs with numbers below 1000 and known pri-miRNA information (“pri-miRNA”).

First, we examined how transcription correlated with miRNA expression in human cells (dataset information in Supplementary Table 1). With miRNA gene structures including the promoters and transcribed sequences often unknown, we used arbitrarily expanded regions centered upon pre-miRNAs to search for their overlaps with Pol2 binding or mRNAs, and then correlated miRNA expression to the retrieved Pol2 ChIP-seq or RNA-seq signals (see section “Materials and Methods”). **Figure 1** shows the Spearman rank correlation coefficients (square symbols) with ChIP-seq analyses in the 11 human cell samples, with numeric data including the additional, sample sizes *N* and *p*-values provided in Supplementary Table 4. Consistent with previous results (Graves and Zeng, 2012), some cell lines including HepG2, GM12878, HeLa, and HCT116 exhibited no significant, positive correlations, while others had positive, weak but significant correlations. In general, the longer the miRNA genomic segments used for overlaps, the more positive the correlations, supporting the prediction that miRNA genes are expansive.

**FIGURE 2:**
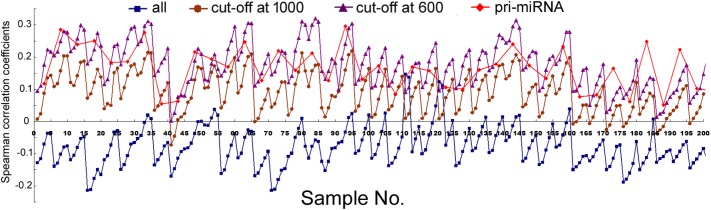
Spearman correlations between miRNA expression and mRNA expression in human cells. Corresponding to the data of Supplementary Table 5, the *y*-axis shows the correlation coefficients, and the *x*-axis represents the 41 cell samples, with 1, 2, 5, 10, and 20 kb genomic extensions from both 5′ and 3′ ends of the pre-miRNAs used for overlap searches. Different miRNA sample sets are indicated by symbols and colors as described in **Figure 1**.

We next compared mRNA and miRNA expression in 41 human cell samples. Surprisingly, most of the correlations were negative, albeit very weak and variable among different cells (**Figure 2**, squares and Supplementary Table 5). Similarly as shown in **Figure 1**, setting larger the miRNA genomic segments used to search for overlaps increased the correlations with mRNA expression.

### Correlations Between Transcriptional Activity and the Expression of Subsets of miRNAs in Human Cells

**FIGURE 3:**
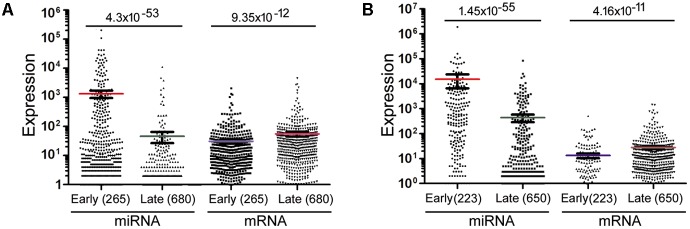
Mann–Whitney *U* test to compare miRNA and mRNA expression in representative samples. **(A)** miRNAs and mRNAs in the GM12878 10 kb data were separated into two groups (“early” and “late”) based on a miRNA cut-off at 600. Spots represent individual RNAs, means and standard errors of means shown as bars, and sample sizes in parentheses. The *p*-values of Mann–Whitney *U* test are shown on top. **(B)** miRNAs and mRNAs in the mouse bladder 2 kb dataset were separated into two groups (“early” and “late”) based on a miRNA cut-off at 400. Labelings are the same as in **(A)**.

The data in **Figure 2** would suggest that transcription plays no or even a negative role in orchestrating differential miRNA expression. This conclusion is counter-intuitive: even though DROSHA processing degrades pri-miRNAs, it is not expected to completely override the effects of transcription. Nonetheless, it has been pointed out that the database likely contains wrongly annotated miRNAs, whose inclusion could obscure the regulation of genuine miRNAs, and/or different miRNAs might be regulated differently (Chiang et al., 2010; Feng et al., 2011; Mitiushkina et al., 2014; Chang et al., 2015). Prime candidates are those RNAs that were expressed at a low level, discovered and added late to the miRBase, hence named with a high number. To test this possibility, we excluded miRNAs named above a certain threshold, e.g., 1000, 800, and 600, and then re-examined the data. This treatment almost invariably increased Spearman correlations, and the lower the threshold, the more positive the correlation coefficients (**Figures 1, 2** and Supplementary Tables 4, 5). For example, using a cut-off at 600, essentially all the correlation coefficients with mRNAs are positive, and most are in the range from 0.1 to 0.3, with *p* < 0.05 (**Figure 2** and Supplementary Table 5). As expected, the excluded miRNAs correlated negatively with their associated mRNA expression (data not shown). ChIP-seq analysis revealed the same, upward trend (**Figure 1**), although the correlation coefficients fluctuated more widely, probably due, in part, to a low number of miRNAs remaining after cut-offs in certain cell lines (Supplementary Table 4). The increases in Spearman correlations could be a statistical quirk or have a biological explanation. To shed more lights on the mechanism, therefore, we divided miRNAs and their linked mRNAs into two groups, “early” and “late,” according to the cut-offs, and compared their respective expression levels. The “early” group had higher miRNA expression but lower mRNA expression (**Figure 3A**). Thus, the later-discovered “miRNAs” are themselves poorly expressed even though transcription around them is stronger, the major contribution to the negative correlations when all miRNAs were considered (**Figure 2**).

In several human and mouse cell lines, hundreds of pri-miRNAs have been experimentally determined (Chang et al., 2015). Thus, we could also examine only those miRNAs using their available pri-miRNA information. When we limited the analyses to this subset of miRNAs, with an additional numbering cut-off at 1000, because such RNAs are also more likely genuine miRNAs, we found that their correlations with mRNA expression, and to a lesser extent, Pol2 binding, increased as well (**Figures 1, 2**, diamond symbols and Supplementary Tables 4, 5). For example, while analyzing all the miRNAs yielded almost consistently negative correlations with mRNA expression in the 41 human cell samples, with the pri-miRNA filter, all 41 samples yielded positive correlations, 28 of which had *p* < 0.05 (**Figure 2** and Supplementary Table 5).

**FIGURE 4:**
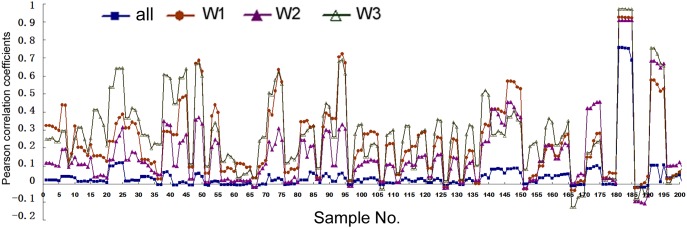
Pearson correlation coefficients between miRNA expression and mRNA expression in human cells. Corresponding to the data of Supplementary Table 6, the *y*-axis shows the correlation coefficients, and the *x*-axis represents the 41 cell samples, each with five different genomic extensions from pre-miRNAs used for overlap searches. Symbols and colors represent various analyses: all the miRNAs in miRBase, not weighted (“all”), and weighted with the three different weighting factors (“W1,” “W2,” “W3”). W1 uses total deep sequencing reads of the whole miRNA stem-loops as the weighting factors, W2 uses the normalized reads per million, and W3 uses the sequencing reads of mature miRNAs (both the 5p and 3p).

Analyses above used artificial cut-offs and experimental pri-miRNA information to stratify human miRNAs. A third, complementary approach is to use publicly available expression data to weight all the miRNAs: if a miRNA is found at a high level overall, it would be given more weight in correlation studies since it is more likely to be a “true” miRNA than a miRNA present at a lower level. We used the existing expression data in miRBase to establish three weighting parameters (see section “Materials and Methods”) and then re-performed correlation studies. With these larger, weighted datasets SPSS could calculated only the Pearson correlations (**Figure 4** and Supplementary Tables 6, 7). **Figure 4** shows the correlations between mRNA and miRNA expression. Without weighting, Pearson correlations are mostly positive but small, with *p* > 0.05 (**Figure 4**, square symbols and Supplementary Table 6). The only exception is for H1-hESC, whose results were skewed by the extremely high expression of the *miR-302* family members and their pri-miRNA. The three weighting factors gave slightly different correlation coefficients, but all three almost universally and greatly increased Pearson correlations (**Figure 4**). Weighting also generally increased the Pearson correlations between miRNA expression and Pol2 binding (Supplementary Table 7).

Taken together, our results suggested that transcription indeed regulated global, differential miRNA expression in human cells, although the effects were modest and variable among different samples. Because 1 kb extensions gave similar results as 2 kb extensions, and longer extensions enhanced correlations (**Figures 1, 2**), we would use 2, 5, 10, and 20 kb extensions (from both the 5′ and 3′ sides) to search for overlapping Pol2 and mRNA signals hereafter.

### Correlations Between Transcription and miRNA Expression in Human Tissues

**FIGURE 5:**
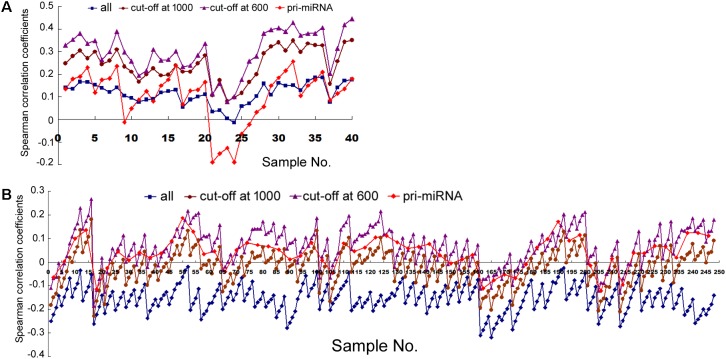
Spearman correlations between transcription and mRNA expression in human tissues. **(A)** Correlation coefficients of miRNA expression and the overlapping Pol2 ChIP-seq signals, corresponding to the data in Supplementary Table 8. The *y*-axis shows the correlation coefficients, and the *x*-axis represents the 10 human tissues, each with 2, 5, 10, and 20 kb genomic extensions from both 5′ and 3′ ends of the pre-miRNAs or pri-miRNAs used for overlap searches. miRNA sample sets are represented by symbols and colors as described in **Figure 1**. **(B)** Correlation coefficients of mRNA and miRNA expression, corresponding to the data in Supplementary Table 9. The *y*-axis shows the correlation coefficients, and the *x*-axis represents the 62 human tissues. Symbols and colors depict miRNA gene sets as in **(A)**.

Next we examined human tissues or organs, apparently from two male and two female, adult individuals (Supplementary Table 2). Comparing Pol2 occupancy and all miRNA expression in 10 tissue samples yielded mostly positive and weak correlations, which were elevated by the cut-off filters as well as by weighting; applying the pri-miRNA filter gave more variable results (**Figure 5A** and Supplementary Table 8). These data are broadly consistent with those obtained in human cells (**Figure 1** and Supplementary Tables 4, 7).

Analyzing mRNA expression and the expression of all the miRNAs in 62 human tissues yielded negative Spearman correlations usually between -0.1 and -0.3, with *p* < 0.05, and, again, the longer the miRNA genomic segments, the less negative the correlations (**Figure 5B**, square symbols and Supplementary Table 9). When we applied the arbitrary number thresholds to examine miRNA subsets, correlations gradually turned less negative and over 50% eventually became positive (**Figure 5B** and Supplementary Table 9). If we considered only those miRNAs whose pri-miRNAs had been experimentally tested (along with a number cut-off at 1000), among the 62 human samples, 14 had negative correlations, 48 positive, 4 of which had *p* < 0.05 (**Figure 5B**, diamond symbols and Supplementary Table 9). Thus, applying the pri-miRNA filter increased correlations compared to the all miRNA group and even the cut-off at 1000 group (**Figure 5B**). Like in human cells, weighting also resulted in typically higher Pearson correlations in human tissues (Supplementary Table 10). Overall, the human tissues exhibited the same patterns and trends upon various analyses as human cells, albeit starting from a more negative base and ending at less positive correlation coefficients.

### Correlations Between mRNA and miRNA Expression in Mouse Tissues

**FIGURE 6:**
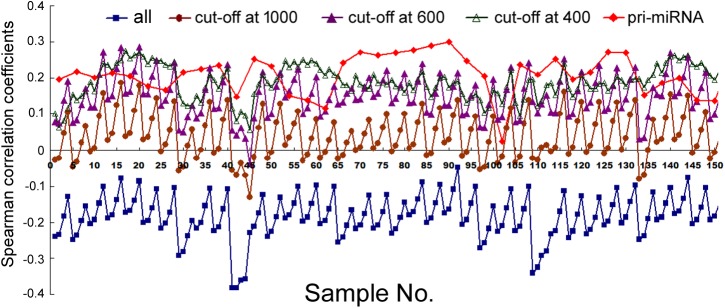
Spearman correlations between miRNA and mRNA expression in mouse tissues. Corresponding to the data of Supplementary Table 11, the *y*-axis shows the correlation coefficients, and the *x*-axis represents the 40 mouse tissues, each with 2, 5, 10, and 20 kb genomic extensions from both 5′ and 3′ ends of the pre-miRNAs used for overlap searches. miRNA gene sets are represented by symbols and colors similarly as described in **Figure 1**.

Lastly, we compared mRNA and miRNA expression in 40 mouse embryonic and postnatal day 0 tissues (Supplementary Table 3). These tissues had Spearman correlations ranging from -0.049 to -0.39, and generally the larger the miRNA genomic segments, the less negative the correlations (**Figure 6**, square symbols and Supplementary Table 11). If we applied the miRNA name cut-offs, e.g., 1000, 800, 600, 500, and 400, to consider the likely bona fide miRNAs, Spearman correlations turned positive very quickly; e.g., at the threshold of 400, all correlation coefficients were positive and mostly between 0.1 and 0.3 (**Figure 6**, hollow triangle symbols and Supplementary Table 11). Using known mouse pri-miRNAs and a cut-off at 1000 as a filter, all 40 mouse tissues had positive Spearman correlations, 39 of them with *p* < 0.05 (**Figure 6**, diamond symbols and Supplementary Table 11). When we directly compared the expression of miRNAs included with the cut-offs with that of the excluded miRNAs, as well as the expression of their associated mRNAs, the included miRNAs were again better expressed than the excluded, while the corresponding mRNAs showed the opposite relationship (**Figure 3B**). All these data closely mimicked those obtained in human cells, and, to a lesser extent, those in human tissues. We had further considered only those miRNAs conserved in both humans and mice. Their correlations were also higher than if all miRNAs were included, although the improvements were not significantly greater than the miRNA number cut-off treatments (data not shown). Lastly, applying weighting to mouse miRNAs yielded divergent results: the trend persisted that weighting typically elevated the Pearson correlations, but there were more exceptions than in human samples, chiefly because the three weighting factors gave dissimilar correlation coefficients (Supplementary Table 12).

### Correlations Between Replicate ENCODE Datasets

**FIGURE 7:**
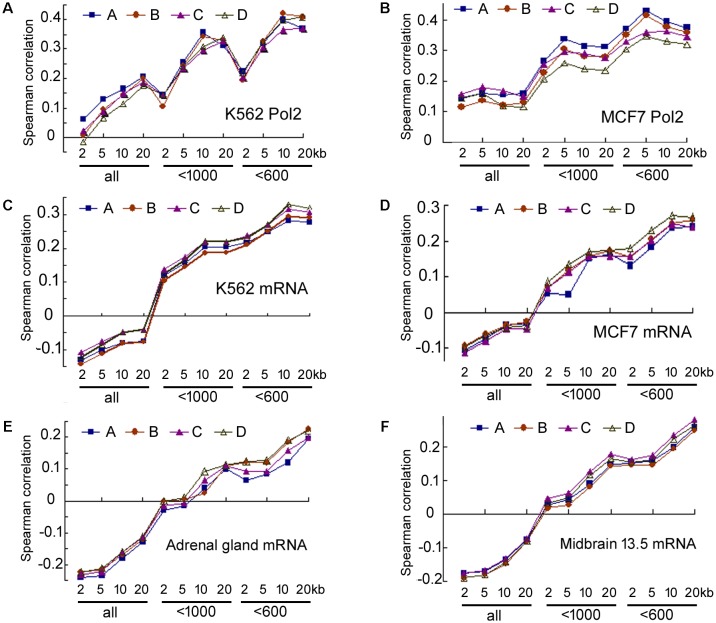
Spearman correlations between duplicate ENCODE datasets. Correspond to the data in Supplementary Table 13. **(A)** Correlations between duplicate Pol2 ChIP-seq and duplicate miRNA datasets in K562 cells. The *y*-axis shows Spearman correlation coefficients. The *x*-axis lists groups of miRNAs and the genomic regions used to search for the overlaps: 2, 5, 10, and 20 kb: extensions from the 5′ and 3′ ends of pre-miRNAs; “all”: all miRBase entries, “<1000”: miRNA number cut-off at 1000, “<600”: cut-off at 600. A, B, C, D: correlations between the four combinations of duplicate datasets (Supplementary Table 13). A is the same one shown in **Figure 1**. **(B)** Correlations between duplicate Pol2 ChIP-seq and duplicate miRNA datasets in MCF7 cells. Labelings are the same as in **(A)**. **(C–F)** Correlations between duplicate mRNA and duplicate miRNA datasets in K562, MCF7, mouse adrenal gland, mouse midbrain embryonic day 13.5 tissues, respectively. Labelings are similar to those in **(A)**.

Our analyses of the data in human cells, human tissues, and moue tissues all pointed to a weak correlation between transcriptional activity and miRNA maturation. But correlation might be underestimated due to experimental errors in the quantification of DNA binding and RNAs, which can be accounted for by calculating the correlations between replicate data (Csardi et al., 2015). Human cells and mouse tissues, but not human tissues, have replicate ENCODE datasets. We thus performed additional analyses in human cells and mouse tissues (Csardi et al., 2015), and six examples are shown in **Figure 7** and Supplementary Table 13. Correlations between duplicate ChIP-seq datasets and duplicate miRNA datasets of human K562 cells are similar (**Figure 7A**), so are those of the human MCF7 cells (**Figure 7B**) and the correlations for mRNA and miRNA expression in representative human cells and mouse tissues (**Figures 7C–F**). The Spearman correlations between duplicate datasets are between 0.84 and 0.98, indicating a high degree of reproducibility, as noted before (ENCODE Project Consortium, 2012; Landt et al., 2012; Ballouz and Gillis, 2016). Consequently, experimental noise correction did not significantly improve correlations. For example, the correlations between 20 kb Pol2 occupancy and miRNA expression in K562 cells are 0.177–0.206 (Supplementary Table 13), and only 0.22 after correction (Csardi et al., 2015). Analyzing more replicates did not offer dramatic improvements (data not shown), and it is practically impossible for the negative correlations between total miRNA and mRNA expression in human cells and mouse tissues to become positive after noise correction (**Figures 7C–F**).

The degradation of mammalian miRNAs has received relatively little attention, but there are variations in their stability in the literature (Sethi and Lukiw, 2009; Bail et al., 2010; Gantier et al., 2011; Ruegger and Grosshans, 2012; Pogue et al., 2014; Marzi et al., 2016). For example, while most miRNAs might have half-lives of over 48 h, a few miRNAs have relatively fast turnover rates, e.g., with a half-life of less than 5 h in 3T9 mouse fibroblasts (Marzi et al., 2016). When we considered miRNA stability in our studies of mouse tissues, we found that the less stable miRNAs tended to have lower correlations with mRNA expression, compared to the stable miRNAs or all the miRNAs, even though the effects were minor (data not shown). Because the identified unstable miRNAs number only 20–30 (Marzi et al., 2016), the contribution of miRNA stability to miRNA expression requires more data and studies.

### Hierarchical Cluster Analysis of the Correlations in the Human and Mouse Tissues

**FIGURE 8:**
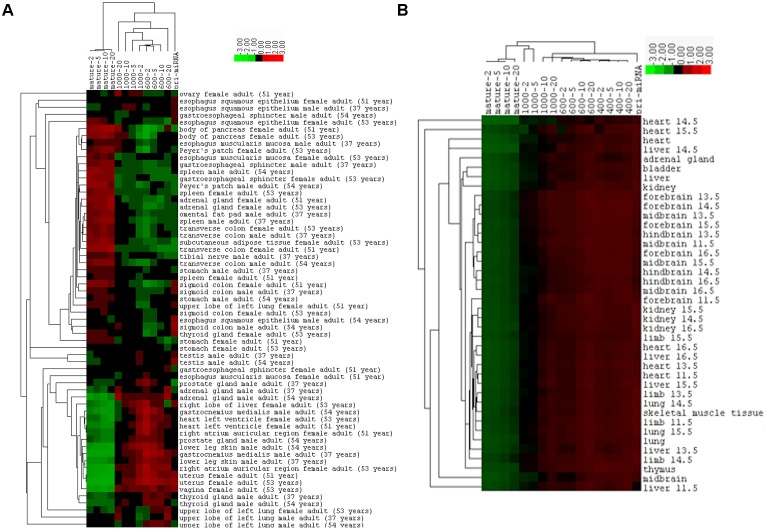
Hierarchical cluster analysis based on Spearman correlations between miRNA and mRNA expression. **(A)** Clustering of the human tissues, according to the data of Supplementary Table 9 and as exactly shown in **Figure 5B**. **(B)** Clustering of mouse tissues, according to the data of Supplementary Table 11 and as shown in **Figure 6**.

The correlations between miRNA expression and transcriptional activity such as mRNA expression are not only weak but also variable among samples, which might be due to the intrinsic differences among the individuals, cell types, tissues, or due to their unequal data qualities. To understand the reasons behind the variability, we performed hierarchical cluster analysis to group the human and mouse tissues or organs based on their Spearman correlation coefficients. As shown in **Figure 8A** for the human samples, the same tissues from males tend to cluster together, so do those from females, but all the same tissues from both males and females are never the closest neighbors. This could be due to experimental noise or reflect real gender differences. Mouse samples exhibited the same pattern: all except one of the brain tissues are grouped together, while liver samples are all separated (**Figure 8B**). Correlation coefficients of mouse samples appear more homogeneous than those of human samples, so the separated mouse samples would still be quite similar (**Figure 8**). Overall, our results suggest that both innate biological differences and sample handling differences contribute to the variations in correlation coefficients.

## Discussion

While a great deal of efforts have been made to understand how transcription regulates protein expression at a large level, in particular the relationship between steady-state mRNA abundance and protein abundance, little is known about how much transcription determines the levels of ultimate gene products when they are RNAs instead of proteins. The default assumption is that transcription exerts a predominant role, although evidence suggested that it might not always be true (Graves and Zeng, 2012; Conrad et al., 2014; Marzi et al., 2016). Here we used the miRNA system as an example to study how transcription regulates RNA expression globally. Our main conclusion is that transcription contributes only modestly to differential miRNA expression in humans and mice.

Nascent nuclear transcripts including pre-mRNAs and pri-miRNAs are generally short-lived, present at low levels, and difficult to quantify precisely, due to co-transcriptional processing such as splicing and cleavage by DROSHA. Consequently, we have used Pol2 occupancy and mRNA expression to approximate transcription around miRNA genomic loci. Pol2 occupancy correlates positively with miRNA expression in a majority of human cells and tissues (**Figures 1, 5A**). mRNA expression, on the other hand, correlates negatively with the expression of all the miRNAs deposited in miRBase in human cells, human tissues, and mouse tissues (**Figures 1, 5B, 6**). Nevertheless, when we applied number and pri-miRNA filters to examine subsets of miRNAs, Spearman correlations turn positive almost uniformly (**Figures 1, 2, 5–7**). The increase in correlation has a biological basis, and filtering is justified because many of the later-annotated “miRNAs” in miRBase tend to be poorly conserved and expressed, whose pri-miRNAs are poorly processed by DROSHA (Feng et al., 2011). Transcription around these genes is generally high, despite low miRNA maturation (**Figure 3**). Consistent with these analyses, adding weights to the more abundant, likely miRNAs also increases correlation to transcription (**Figure 4**). Our current study, therefore, reinforces the notion that either many of these “miRNAs” are mischaracterized, or they are regulated differently compared to the canonical miRNAs (Chiang et al., 2010; Feng et al., 2011; Mitiushkina et al., 2014; Chang et al., 2015). One possibility is that some miRNAs may be transcribed by RNA polymerase III. We have examined the relevant ENCODE ChIP-seq datasets but found few overlaps with miRNA genes, almost all of which are also already bound by Pol2 (data not shown). Thus, current data do not support an alternative hypothesis that RNA polymerase III regulates a large number of miRNA genes. Other possibilities include, for example, that transcription plays a more prominent role in the maturation of canonical miRNAs, while processing in that of the non-canonical miRNAs.

Even though our results establish that transcription positively regulates global miRNA expression, they also suggest that the contribution is relatively small, with Spearman rank correlation coefficients rarely above 0.4 (ChIP-seq correlations) or 0.3 (mRNA correlations) even after threshold application. In theory, the contributions (ρ^2^) by transcription, processing, and stability should add up to 1. Unfortunately, due to the incompleteness of data and the presence of experimental noise and systematic noise, one does not know where the current ceiling is. But even if a coefficient of 0.4 here signals a rate-limiting contribution, an improbable proposition, many other human and mouse samples still have lower coefficients. A more plausible conclusion is that, in contrast to most assumptions, transcription does not play a predominant role in setting relative miRNA expression levels globally. This infers that post-transcriptional events including miRNA processing and degradation must exert critical, regulatory roles. For example, a previous study showed a correlation coefficient of 0.51 between selective pri-miRNA processing by DROSHA and human miRNA expression (Feng et al., 2011), although the value is not directly comparable to the correlations here. As a special case, miRNAs in clusters, presumably transcribed identically, have dissimilar expression, likewise suggesting the importance of miRNA processing (Chaulk et al., 2011; Feng et al., 2011; Marzi et al., 2016). It is also essential to study how degradation contributes to the regulation of miRNA expression and function. Different miRNAs may have different half-lives, and the same miRNAs may have different stabilities in different cell types (Sethi and Lukiw, 2009; Bail et al., 2010; Gantier et al., 2011; Ruegger and Grosshans, 2012; Pogue et al., 2014; Marzi et al., 2016). We would like to note, however, that this study has examined a wide range of human and mouse cell types and tissues and found overall similar correlation coefficients. It will also be interesting to examine other non-coding RNAs in an analogous manner (Engreitz et al., 2016).

While our work represents the most comprehensive analyses of the global relationship between transcription and non-coding RNA expression thus far, future studies with better modeling and data can improve on a number of fronts. One is that we applied the same miRNA genomic or pri-miRNA information to all the human cells, human tissues, and mouse tissues. The actual situation is obviously more complex. The second is that correlations vary among samples, likely influenced by their intrinsic biological differences and unequal data qualities as well (**Figure 8**). For example, Pol2 peaks overlap with a much lower number of miRNAs in some human cells than in others (Supplementary Table 4), and human tissues lack replicate ENCODE datasets. The third is that correlation will benefit from a better “filter” or weighting factor to separate true miRNAs from irrelevant RNAs. Number thresholds are crude and arbitrary. The three weighting parameters from miRBase are less subjective and cover a wide range of biological samples, but they produce variable results, especially in mouse tissues (Supplementary Table 12). This is likely because the parameters are built on a large number of studies from many different laboratories with minimal control over quality, standardization, and consistency. Lastly, miRNA processing and degradation need to be incorporated to better understand how miRNAs are regulated at the genome level. One should also note that even if transcription does not have an oversized contribution in determining differential miRNA levels globally, its role in regulating the expression of individual miRNAs in a temporally and spatially specific manner or in response to other stimuli is well known and of paramount biological significance.

## Author Contributions

YZ designed the studies. XZ, SH, JS, ZX, WL, and YZ performed the data analyses. All the authors contributed to manuscript preparation.

## Conflict of Interest Statement

The authors declare that the research was conducted in the absence of any commercial or financial relationships that could be construed as a potential conflict of interest.

## Footnotes

1 https://www.encodeproject.org

2 https://usegalaxy.org
